# The stable component of maternal depressive symptoms predicts offspring emotional and behavioral symptoms: a 9-years longitudinal study

**DOI:** 10.1186/s40359-020-00496-0

**Published:** 2020-12-01

**Authors:** L. Cerniglia, F. Dentale, R. Tambelli, L. Murray, P. Cooper, S. Cimino

**Affiliations:** 1 Faculty of Psychology, International Telematic University Uninettuno, Rome, Italy; 2 Department of Clinical and Dynamic Psychology, Sapienza, University of Rome, Via dei Marsi, 78, 00186 Rome, Italy; 3 School of Psychology and Clinical Language Sciences, University of Reading, Reading, UK

**Keywords:** Maternal depression, LST, Children’s internalizing/externalizing, Dysregulation symptoms

## Abstract

**Background:**

Maternal sub-threshold and non-clinical depression and its possible outcomes on offspring internalizing/externalizing symptoms has received growing attention in recent years because of its significant worldwide prevalence.

**Methods:**

Through a Latent State-Trait Analysis approach (LST), this longitudinal study aimed to identify a stable component of non-clinical maternal depression across a temporal interval of 6 years (measured through the Symptom Check-List-90/R) and to determine the effect of this component on children’s emotional and behavioral functioning (measured through the Child Behaviour Check-List) at age 12 years.

**Results:**

LST analysis showed that maternal depressive symptoms tended to remain stable within individuals across 6 years of observation strongly contributing to children’s internalizing/externalizing and dysregulation symptoms.

**Conclusions:**

The current longitudinal analysis of maternal and child data revealed that a stable component of maternal depressive symptoms reliably predicted a wide range of child emotional and behavioral symptoms at 12 years of age.

## Background

Clinical depression is a major contributor to the disease burden worldwide and constitutes a significant economic burden for communities [1], with a life-time prevalence between 20 and 25% in women and 7–12% in men [2–4]. Although historically psychiatric nosology has regarded major depression as a distinctive disorder [5, 6], more recent research has indicated that depression should be conceptualized as existing on a continuum [7]. Indeed, sub-threshold and non-clinical depression has received growing attention in recent years because of its significant prevalence (population studies have showed rates ranging from 1.4 to 17.2%) [8].

Although literature demonstrated that emotional and behavioral problems in children are predicted by several risk factors (e.g. child biology, child cognitive functioning, family context, school context) [9], depression in mothers has attracted considerable research and clinical attention over recent decades, largely because of evidence of its adverse effect on the mother–child relationship and child developmental outcome [10, 11]. However, most of this evidence comes from studies of mothers with a psychiatric diagnosis of depressive disorder, particularly peri-partum depression (with the exception of high quality studies such as the UK Avon Longitudinal Study of Parents and Children study [12], the Dutch Generation R study [13], the Canadian National Longitudinal Survey of Children and Youth [14], and the US National Institute of Child Health and Human Development study [15]), largely, or entirely, excluding mothers with sub-clinical and non-diagnosed presentations whose prevalence in the general population, as noted, is substantial, raising the question of the possible effect on mothers and infants of depression in this non-clinical form.

Research conducted within the theoretical and clinical framework of Developmental Psychopathology [16] has widely demonstrated that mothers’ depression predicts children’s internalizing and externalizing problems [17]. A number of processes may operate in these associations. Children’s symptoms could be affected by impaired maternal caregiving capacities and associated poor mother–child interactions [18]. Children could be exposed to stressful environments and lack of social support that negatively influence child behavior [11]. Mothers and their offspring could share genetic characteristics associated with psychopathology that account, at least in part, for both the maternal depression and the child emotional or behavioral disturbance [10, 19]. Notably, the intergenerational maladaptive influence on children of maternal depression has been associated with the chronicity of maternal symptom (even when such symptoms were at subclinical levels) [20–22].

As well as overall internalizing and externalizing symptoms, more recent literature has shown that maternal depression is associated with emotional and behavioral dysregulation in children [23, 24]. As several scholars have indicated, while adaptive emotion regulation processes support the formation of the self, a personal sense of self-efficacy, and social skills, emotional/behavioral dysregulation can be associated with hypervigilance, anxiety, inhibition, and attachment insecurity [25].

Although maternal depression and its predictive role for the onset of emotional-behavioral problems in children has been widely studied, research has rarely been longitudinal, has generally focused on small clinical samples, and has made assessments over a limited periods of time (usually from early childhood to toddlerhood) [11, 14, 26]. An exception to this is the recent longitudinal study using the AVON birth cohort data [27], which assessed children up to the age of 18 years. While this study was concerned with the impact of varying levels of severity of identified maternal depression, rather than continuous community sample data, one finding of particular note was the importance of persistent depression in predicting adverse child outcomes. In this line of reasoning, an interesting further research aim would be to investigate if stable sub-clinical depression in mothers from early to middle childhood can influence children’s outcomes on different psychopathological symptoms. In order to explore a similar research question, longitudinal data with multiple occasion of measurement for both mothers and children across different ages of children (e.g., 2–8 years) are necessary. With this kind of longitudinal multi-occasion dataset, it would be possible to apply specific analytic strategy, such as the Latent State-Trait [LST; 28] analysis, that allow to disentangle latent occasion-specific and stable components of a target measure. By means of this analysis, the observed variance of a test can be separated into trait-specific and systematic occasion-specific components by testing participants at different occasions of measurement (for an introduction to LST analysis, see [28]). LST analysis has already been successfully applied in the area of the positive and negative affect measurement [29], and also in the field of developmental psychopathology [30]. For the aims of the present study, it is of particular interest the possibility to estimate the proportion of stable variance of maternal depression with respect of occasion-specific variance, and also the predictive power of maternal stable component on children’s psychopathological outcomes.

In this view, the present study is aimed to apply the Latent State-Trait [LST; 28] analysis across a temporal interval of 6 years (at age 2, 5, 8 of the children) in order to disentangle stable, occasion, method and error components of maternal depression as measured with the SCL-90/R dedicated scale (see in the Analytic Strategy section for a more detailed description of the specific models we used). Importantly for the present study, the application of LST models [e.g., 31, 32] allowed us to estimate the stable component of maternal depression and, successively, testing its predictive power on children’s symptoms at age 12 (as measured with CBCL scales). In particular, consistently with the transmission multifinality approach [33] and with the studies of Kim et al. [34], the unique predictive contribution of the stable component of maternal depression on children’s internalizing/externalizing and dysregulation symptoms at age 12 was investigated also when the corresponding symptoms at age 8 were controlled for.

## Method

### Participants and procedure

This work is part of a larger study, begun in 2010 as a screening program, in collaboration with public and private schools in Italy, for the evaluation of psychopathological risk in community samples—in particular in mothers and children. The study protocol was approved by the Ethical Committee of the Psychology Faculty of Sapienza, University of Rome, in accordance with the guidelines approved in Helsinki Declaration. The dataset is visible here: http://dx.doi.org/10.17632/bx62rd3tc2.2. Sample assessment was conducted over four waves [Occasion 1 (O1), Occasion 2 (O2), Occasion 3 (O3), Occasion 4 (O4) corresponding to 2, 5, 8, 12 years of child age]. A group of psychologists administered the measures at the children’s schools after contacting headmasters and receiving their agreement to the participation of the school in the study. Mothers were contacted and informed about the aim of the study before its start and aggregated results were communicated to parents after its end. The initial sample comprised 272 2-years old children and their mothers. The exclusion criteria were as follows: thirty-two dyads were excluded at O1, due to children’s organic or physical problems. Thirty dyads were excluded from the present sample because mothers reported psychopathological diagnoses (eleven mothers with Major Depression Disorder; fourteen mothers with Anxiety Disorder; five mothers with Borderline Personality Disorder). Fifteen dyads were excluded due to the mothers’ inability or refusal to participate in the subsequent waves of assessment after the O1 assessment. Finally, thirty-five dyads were excluded from the current study due to incomplete or missing data at one or more assessment waves. The sample for the current study therefore comprised 160 mothers and children [mean child age in years at O1, O2, O3 and O4 respectively were 2.51 (SD = 1.22), 5.12 (SD = 1.25), 8.32 (SD = 1.43), and 11.52 (SD = 1.58)]. Considering all data analyses we conducted, the number of participants we recruited (n = 160) was sufficient to obtain a statistical power of .80 for small to medium effect size and a critical alpha of .05 (two tailed). All mothers signed written consent and there was little data loss over the 10-year data collection period. As indicated by the socio-demographic questionnaire at O1, 92% of mothers and children belonged to intact families with average socio-economic status (25,000–30,000 Euros per year) and educational level (high school or university) [35].

On all four occasions of measurement, mothers filled out the Symptom Checklist or SCL-90/R [36] to provide an index of the level of their own depressive symptoms, and the Child Behavior checklist or CBCL scales [37] to provide a wide assessment of child emotional and behavioral symptoms.

### Measures

#### Maternal depressive symptoms

To assess maternal depressive symptoms, the SCL-90/R [36] was completed by the mothers at all four assessment waves. The SCL is a 90-item self-report symptom inventory measuring psychological symptoms and psychological distress. It encompasses nine main symptom dimensions, but for the current study we only considered the Depression subscale that is formed by 13 items. In this study, all the mothers who reported psychopathological diagnoses (including Major Depression) Disorder were excluded from the sample. The SCL-90/R has shown a good internal consistency in adults in clinical and community samples (For the Italian version see Prunas et al.) [38]. For the current study, in order to estimate the stable component of maternal depressive symptoms and test its unique contribution in the prediction of children’s symptoms, only O1, O2 and O3 measurements were included for the SCL-90/R.

#### Children emotional-behavioral functioning

The mothers also completed the CBCL 1,5-5 at T1 and T2 [33] and the CBCL 6-18 at assessments waves O3 and O4. The CBCL 6-18 is a questionnaire completed by caregivers to assess children’s abilities and their specific behavioral/emotional characteristics. This instrument comprises eight specific subscales (i.e., Anxious/Depressed, Withdrawn/Depressed, Somatic Complaints, Social Problems, Thought Problems, Attention Problems, Rule-Breaking Behavior, and Aggressive Behavior), as well as two global scales: Internalizing Problems (consisting of Anxious/Depressed, Withdrawn/Depressed, and Somatic Complaints subscales), and Externalizing Problems (consisting of Rule-Breaking Behavior and Aggressive Behavior subscales). The criterion-related validity of both versions of the CBCL is supported by the ability of the CBCL’s quantitative scale scores to discriminate between demographically matched, referred and non-referred children [34]. In the present study, the Italian validated version of both general and specific scales of CBCL 6-18 [39] were used, along with the Dysregulated Profile (i.e., an aggregate of the Anxious/Depressed, Aggressive Behavior, Attention Problems subscales) [34]. For the present study only the CBCL 6-18 version (O3 and O4) CBCL data were used.

#### Analytic strategy

The analytic strategy of the present study included three different steps, that are (1) preliminary analyses; (2) applying the LST analysis on longitudinal maternal depression data in order to extract the maternal depression stable component; (3) evaluation of the predictive effects of maternal depression stable component on children’s symptoms at age 12, controlling for the symptom levels at age 8.

(1) With regards to maternal depression, a series of preliminary analyses were conducted (see Tables 1, 2) to provide descriptive statistics and intercorrelations of the test-halves of SCL-90 dedicated scale, along with an estimate of the its internal consistency. With regards to the children, descriptive statistics and internal consistency of CBCL symptoms scales were reported (see Table 3) both for time point 3 (at age 8) and 4 (at age 12), along with a statistical test of differences between the two occasion of measurement. These preliminary analyses were conducted using SPSS 25 package;

**Table 1:** Descriptive statistics of SCL-90/R depression test-halves

	Mean	SD	Skewness	Kurtosis	R_tt_
SCLDEP1_I	1.65	1.22	.11	− 1.55	.96
SCLDEP2_I	1.59	1.31	.10	− 1.70	
SCLDEP1_II	1.58	1.34	.07	− 1.74	.97
SCLDEP2_II	1.51	1.31	.08	− 1.80	
SCLDEP1_III	1.46	1.38	.13	− 1.80	.97
SCLDEP2_III	1.39	1.31	.15	− 1.77

**Table 2:** Correlations among SCL-90/R depression scale halves

	SCLDEP1_I	SCLDEP2_I	SCLDEP1_II	SCLDEP2_II	SCLDEP1_III	SCLDEP2_III
SCLDEP1_I	1					
SCLDEP2_I	.92**	1				
SCLDEP1_II	.90**	.92**	1			
SCLDEP2_II	.91**	.92**	.95**	1		
SCLDEP1_III	.90**	.91**	.94**	.94**	1	
SCLDEP2_III	.91**	.92**	.93**	.94**	.94**	1

**The correlation is significant at .01 level (two tailed)

**Table 3:** Descriptive statistics (mean, SD, Skewness and Kurtosis) and internal consistency (Cronbach’s alpha) of CBCL scales for the third (outside brackets) and the fourth (inside brackets) occasion of measurement, along with t tests for the mean differences (mean3–mean4) between the two measurement occasions

	Mean3 (Mean4)	SD3 (SD4)	SK3 (SK4)	KU3 (KU4)	α3 (α4)	t (159)	p
ANX	7.24 (7.68)	5.76 (5.80)	− .02 (− .03)	− 1.51 (− 1.50)	.85 (.85)	− 3.48	.00
WITH	4.29 (4.54)	5.34 (3.92)	1.14 (.52)	− .47 (− .79)	.94 (.80)	− .81	.42
SOM	3.75 (4.14)	3.47 (3.90)	.98 (.98)	.10 (.17)	.78 (.81)	− 2.96	.00
INT	15.29 (16.36)	12.49 (12.56)	.38 (.17)	− 1.20 (− 1.37)	.93 (.93)	− 3.29	.00
SOC_PR	4.33 (4.88)	3.73 (4.29)	.68 (.81)	− .34 (.08)	.76 (.80)	− 3.96	.00
TH_PR	4.81 (5.66)	4.20 (5.13)	.84 (1.21)	.59 (2.21)	.77 (.82)	− 5.32	.00
ATT	3.98 (4.32)	3.07 (3.41)	.51 (.54)	.24 (− .25)	.68 (.72)	− 3.54	.00
RU_BR	4.89 (5.63)	4.67 (5.50)	.40 (1.35)	2.61 (2.23)	.80 (.83)	− 4.54	.00
AGGR	6.86 (7.54)	6.16 (6.90)	.21 (1.16)	1.66 (1.35)	.85 (.87)	− 3.74	.00
EXT	11.76 (13.16)	10.53 (12.08)	.31 (1.27)	2.34 (2.00)	.96 (.96)	− 4.47	.00
EM_DYS	6.03 (6.51)	4.67 (5.03)	.46 (.46)	− .22 (− .44)	.89 (.89)	− 4.25	.00

*ANX* anxiety and depression, *WITH* withdrawal and depression, *SOM* somatization, *INT* internalizing disorders, *SOC_PROB* social problems, *TH_PROB* thought problems, *ATT* attention problems, *RU_BR* rule breaking behaviors, *AGGR* aggressive behaviors, *EXT* externalizing behaviors, *EM_DYS* emotional dysregulation

*All correlations are significant at critical at .001 alpha level (two tailed)

(2) In order to estimate the stable and occasion-specific components of maternal depression across three time points (i.e., 2, 5, 8 years of the children), a Latent State-Trait [LST; 28] analysis approach was used. In general LST models allow to disentangle four different latent factors (i.e., stable, occasion-specific, method, and error components) that are assumed to determine the scores of a hypothetic Y measure observed on different longitudinal occasions, allowing for the estimation of four components of variance: (1) consistency [Con(Y) = Var(T)/Var(Y)], that is the proportion of trait variance; (2) occasion specificity [OccSpec(Y) = Var(O)/Var(Y)], that is the proportion of occasion-specific variance; (3) method-specificity [MetSpec(Y) = Var(M)/Var(Y)], that is the proportion of method variance; (4) measurement error [MeasErr(Y) = Var(E)/Var(Y)], that is the proportion of error variance. This decomposition of variance permits the estimation of the reliability of the measure: Rel = 1 − Var(E)/Var(Y) = Con(Y) + OccSpe(Y) + MetSpe(Y), where E represents the measurement error.

**Fig. 1:**
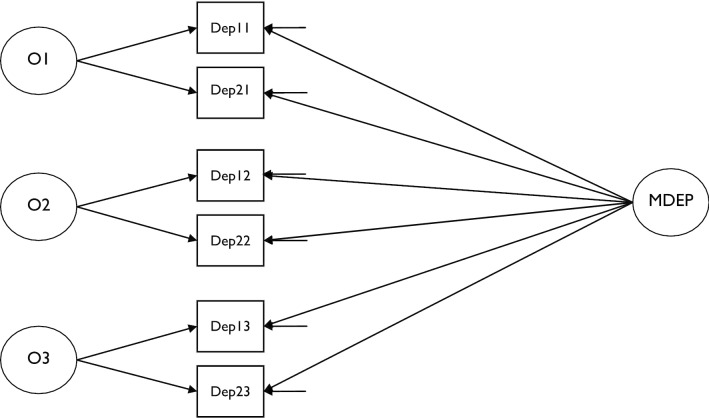
Latent State-Trait model including a general stable component of maternal depression (MDEP) and three different occasion components (O1: at age 2 of the children; O2: at age 5 of the children; O3: at age 8 of the children)

With regards to the present study (see Fig. 1 to see a graphic of the model tested), to estimate occasion latent factors (i.e., O1, O2 and O3), at least two parallel measures of maternal depression for each time points are necessary. For this reason, SCL-90/R depression items were assigned to two different test-halves in such a way as to optimize their conceptual parallelism. As shown in Fig. 1, the stable latent factor of maternal depression was estimated including both test-halves in all three occasion of measurement. Moreover, for each test-half a residual component was estimated representing the measurement error. Not illustrated in the model of Fig. 1 are the method factors (representing the common variance of each test-half across the three occasion of measurement) as the best fitting solution for our data is the No Method (NM) model that does not include method factors [As reported in the results section, the best fitting solution among No Method No Occasions (NMNO), No Method (NM) and all Method minus 1 (M-1) models were tested (see also Fig. 1, and Table 4)]. Since the reliability coefficient estimated using the LST models in this case refers to one test-half only, aggregation equations were applied that may be considered as a generalization of the Spearman–Brown formula [28]. The LST models were conducted using M-Plus 8 software package.

**Table 4:** Results of the latent state-trait (LST) analysis on maternal depression as measured in three occasions of measurement, that are at age 2, 5, 8 of the children)

LST models	Model fit			Latent variances					LST coefficients				
	χ^2^	df	p	Trait	Occasion	Method	Error	Total	Con	OccSpe	MetSpe	Error	Rel
No method no occasion factors (NMNO)	22.76	14	.06	1.32	–	–	.07	1.39	.95	–	–	.05	.95
No method factor (NM)	12.18	13	.51	1.32	.03	–	.06	1.41	.94	.02	–	.04	.96
With method factor (M-1)	12.00	12	.45	1.31	.03	.003	.06	1.41	.94	.02	.002	.04	.96

N = 160

*Con* consistency, *OccSpe* occasion specificity, *MetSpe* method specificity, *Rel* reliability corrected for the total test

**Fig. 2:**
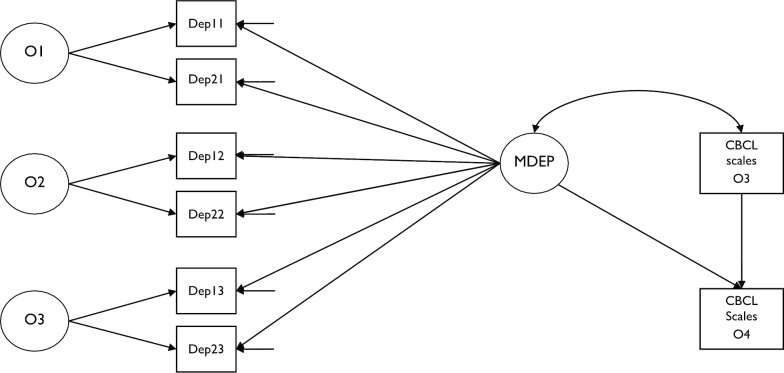
Latent State-Trait (LST) model estimating three occasions components (O1: at age 2 of the children; O2: at age 5 of the children; O3: at age 8 of the children) along with a general stable component of maternal depression (MDEP) included as a predictor of CBCL scales as measured in the fourth occasion of measurement (O4: at age 12 of the children). CBCL scales were also autoregressed on their scores as measured in the third occasion of measurement (O3: at age 8 of the children)

(3) In order to test the predictive power of maternal depression stable component on children’s symptoms, a series of models were analyzed including a factorial portion, based on the LST analysis described above, and a predictive portion, based on a Cross Lagged Panel Model [(CLPM); 30], applied to estimate the effect of maternal depression stable component on children’s symptoms at age 12, controlling for children’s symptoms at age 8 (see Fig. 2, and Tables 5 and 6). These models were conducted using M-Plus 8 software package.

**Table 5:** LST analyses including the stable component of maternal depressive symptoms as predictor, and the fourth observation (at age 12) of Internalizing symptoms (model 1), Externalizing symptoms (model 2) and Emotional Dysregulation (model 3) CBCL scales as criteria

Model		Model fit						Coefficients		Comparison	
LST model	Criterion	χ^2^ (23)	P	RMSEA	CFI	TLI	SRMR	SSCMD	SSCA	Δχ^2^(1)	p
1	INT	25.13	.34	.02	1.00	1.00	.01	.42**	.58**	32.59	.00
2	EXT	32.11	.10	.05	.99	.99	.01	.14*	.84**	7.27	.01
3	EM-DYS	24.44	.38	.02	1.00	1.00	.01	.16**	.82**	11.56	.00

For each model the corresponding CBCL scales as measured in the third observation (at age 8) were included as covariates (see Fig. 2)

*INT* CBCL internalizing scale, *EXT* CBCL externalizing scale, *EM-DYS* CBCL emotional dysregulation scale, *SSCMD* standardized structural coefficient of the stable component of maternal depression, *SSCA* standardized structural coefficient of the autoregression for each criterion, *Δχ*^*2*^*(1)* chi square of the difference with 1 degree of freedom between the model investigated and the same model with the structural coefficient of maternal depression fixed to 0

**Table 6:** Results of eight LST analyses that included maternal depression stable component as predictor, and the fourth observation (at age 12) of children’s Anxiety (model 1), Withdrawal (model 2),Somatization (model 3), Social Problems (model 4), Thought Problems (model 5), Attention Problems (model 6), Rule Breaking Behaviors (model 7), Aggressive Behaviors (model 8) as criteria

Model		Model fit						Coefficients		Comparison	
LST model	Criterion	χ^2^ (23)	p	RMSEA	CFI	TLI	SRMR	SSCMD	SSCA	Δ χ^2^ (1)	p
1	ANX	22.22	.51	.00	1.00	1.00	.01	.17**	.81**	8.51	.00
2	WITH	24.74	.36	.02	1.00	1.00	.01	.76**	.24**	58.91	.00
3	SOM	23.58	.43	.01	1.00	1.00	.01	.16**	.79**	12.52	.00
4	SOC_PR	24.04	.40	.02	1.00	1.00	.01	.19**	.76**	13.13	.00
5	TH_PR	26.15	.29	.03	1.00	1.00	.01	.08	.87**	.54	.47
6	ATT	20.28	.63	.00	1.00	1.00	.01	.13**	.83**	11.71	.00
7	RU_BR	32.94	.08	.05	.99	.99	.01	.16*	.81**	8.97	.00
8	AGGR	29.37	.17	.04	.99	.99	.01	.13*	.84**	8.08	.00

The third observation (at age 8 of the children) of the latter scales was also included as covariates

*ANX* anxiety and depression, *WITH* withdrawal and depression, *SOM* somatization, *INT* internalizing disorders, *SOC_PROB* social problems, *TH_PROB* thought problems, *ATT* attention problems, *RULE_BR* rule breaking behaviors, *AGGR* aggressive behaviors, *SSCMD* standardized structural coefficient of the stable component of maternal depression, *SSCA* standardized structural coefficient of the autoregression for each criterion, *Δχ*^*2*^* (1)* chi square (1 df) between the model investigated and the same model with the structural coefficient formaternal depression fixed to 0 (every Δ χ^2^ are significant and remain significant also after Benjamini–Hochberg correction)

**The structural coefficient is significant at .01 alpha level (two tailed) and remains significant also after Benjamini–Hochberg correction

*The structural coefficient is significant at .05 alpha level (two tailed) and remains significant also after Benjamini–Hochberg correction

## Results

### Descriptive statistics

As clarified before, a first aim of the present study was to apply a Latent State-Trait (LST) analysis approach to extract the stable component of maternal depression, as measured by the SCL-90/R across three different occasions of measurement (respectively at child age 2, 5 and 8 years). In order to analyze LST models, two test-halves of the maternal depression score were computed. Table 1 shows the descriptive statistics of these test-halves and also the internal consistency (i.e., split-half reliability with Spearman–Brown correction) of SCL-90/R maternal depression across the three measurement occasions. As can be seen, all test-halves of maternal depression showed a substantial normal distribution with slight deviations only for kurtosis (< − 1). To assess longitudinal mean differences across the three assessment waves, repeated-measures ANOVAs were conducted, separately for the two test-halves. Results of these analyses showed a significant multivariate effect for both the first test-half [F(2, 158) = 8.39, p < .001] and the second test-half [F(2, 158) = 12.40, p < .001]. In particular, post-hoc Sidak tests revealed that, for both test-halves, the mean scores for maternal depression at both T2 and T3 were significantly lower than those observed at T1 (p < .001 and p < .01 respectively), indicating a significant reduction of maternal depression overtime.

Table 2 reports the intercorrelations among maternal depression test-halves across the different occasions of measurement. Results showed significant and large correlations among all measures and across all observations, a necessary condition for the application of LST models.

With regards to the children’s symptoms, Table 3 shows the descriptive statistics (i.e., mean, SD, skewness and kurtosis) and Crobach’s alpha values of the CBCL general and specific scales, as measured at T3 (at child age of 8 years—see inside brackets) and T4 (at child age of 12 years—see outside brackets) occasions of measurement. As can be seen from Table 3, some moderate deviations from normality (especially at T4) emerged for some scales (i.e., anxiety, withdrawal, internalizing disorders, thought problems, rule breaking, aggressive behaviors, externalizing behaviors), with skewness and kurtosis values not included within ± 1 interval. Interestingly, apart from the withdrawal scale, mean scores of all scales were significantly lower in the third measurement than in the fourth one, (see the last two columns of Table 3 to examine t test values and the corresponding probabilities), indicating that children’s symptoms tended to increase from 8 to 12 years of age (applying non-parametric tests, not assuming normal distribution of the scales, similar results emerged). Considering the large number of statistical comparisons conducted (eleven t tests), a correction was applied to conventional alpha level (.05) using Benjamini–Hochberg procedure. The Benjamini–Hochberg corrected analyses’ did not show substantial differences when compared with the analyses using conventional alpha levels.

### Extracting stable, occasion and error components of maternal depression across three measurement occasions (at age 2, 5 and 8 of the children) applying LST analysis

In order to disentangle consistency, occasion-specificity, and error variance of maternal depression during children’s development, a Latent State-Trait [LST; 28] analysis approach was applied across three different occasions of measurement (i.e., at age 2, 5 and 8 of the children).

As shown in Fig. 1, a No Method (NM) model with three latent occasion factors, one stable factor, no method factors, and six casual error components were analyzed, including SCL-90/R test-halves of depression scale as observed variables. As shown in Table 4, this model was tested and compared with both a model that also included method latent factors to account for the variability due to test-halves differences, and a model that included only the general stable factor of maternal depression with no occasion and no method factors (NMNO). Regarding the former, recently different approaches that account for method effects in LST models were compared using simulation studies and actual data sets [40]. The model with M − 1 method factors [41], that includes one method factor less than methods used in the study, and the model with no method factors (NM), have both shown unbiased parameter estimates, even for more complex models than the one conducted in the present study (≈ 20 estimated parameters), and also for smaller sample sizes (≈ 100). For these reasons, in the present study a comparison among NM, M-1, and NMNO was conducted to identify the best fitting model (see Table 4).

Since some of the measures showed slight deviations from normal distribution, we used maximum likelihood estimation robust to non-normality (MLR). Moreover, we fixed to 1 all factor loadings for each latent factor in order to increase the ratio between number of participants and number of estimated parameters [42]. Finally, we constrained the occasion-specificities and the error variances to be equal.

Results showed that the NM model displayed an adequate fit with a non-significant chi square (see Table 4) and excellent levels for the other indices (RMSEA = .00; CFI = 1.00; TLI = 1.00; SRMR = .01). Similar results were found for the less restricted M-1 model that also revealed a non-significant chi square (see Table 4), and optimal levels for the other indices (RMSEA = .00; CFI = 1.00; TLI = 1.00; SRMR = .01). Finally, the NMNO model, that included only the general depression factor (excluding both occasion and method factors), showed a close to significant chi square (see Table 4), non-optimal levels for the RMSEA (.06), and adequate levels for the other indices (CFI = .99; TLI = .99; SRMR = .01). In order compare the fit of these models, the NM model was compared with both the M-1 and the NMNO ones applying the Δχ^2^ test. Results of these tests revealed that the NM model fitted better than the NMNO one [Δχ^2^(1) = 9.08, p < .01], and fitted similarly to the less parsimonious M-1 model [Δχ^2^(1) = .09, p = .76], indicating that the NM model was the best fitting one. As illustrated in Table 4, consistency was much higher than occasion-specificity, suggesting that maternal depression as measured by the SCL-90/R was highly stable overtime. It is worth noting that, when aggregating results of the two halves, excellent reliability was found for the SCL-90/R depression scale. Since, in the final NM model, for each latent factor all loadings were fixed to 1, and occasion-specificities and the error variances were constrained to be equal, a further statistical test was conducted to evaluate the adequacy of these restrictions. In particular, the NM model was compared to a model that relaxed these restrictions. No significant differences were found [Δχ^2^(3) = 4.32, p = .23], indicating that the more parsimonious restricted model was the best fitting one.

### The stable component of maternal depression as a predictor of children’s symptoms

In order to investigate the predictive power of the stable-component of maternal depression on children’s symptom levels, the NM model was used including two additional observed variables (see Fig. 2): the CBCL scales as measured at O3 (at 8 age of the children) and at O4 (at age 12 of the children). The model proposed in Fig. 2 included both a Latent State-Trait approach (LST) [28]—referring to the factorial estimation of the stable component of maternal depression across the different occasions of measurement—and a Cross Lagged Panel analysis (CLPM) [32]—referring to the path analysis aimed to estimate the effect of maternal depression stable component on children's symptomatic scales at O4 controlling for children's symptomatic scales at O3.

More specifically, in a first set of three LST models, the stable-component of maternal depression estimated at O1, O2, and O3 (i.e., child age 2, 5, and 8 respectively), was included as a predictor, the O4 measure of each of the three general CBCL scale (i.e., Internalizing/Externalizing problems, and Emotional Dysregulation scales) was included as criterion, and the corresponding O3 measure of CBCL scale was included as a control variable. As shown in Table 5, each LST analysis (i.e., 1, 2, 3 models including as criterion, respectively, Internalizing, Externalizing and Emotional Dysregulation scale) fitted the data well, with no significant chi-square, and with adequate levels for the other indices (RMSEA ≤ .05; CFI, TLI ≥ .99; SRMR ≤ .01). Moreover, as expected, the stable-component of maternal depressive symptoms showed significant predictive power for each CBCL general scale at age 12, even when the corresponding CBCL scale at age 8 was controlled for. In particular, as shown in Table 5, in each model both the standardized structural coefficient of maternal depression (SSCMD) and the standardized coefficient of autoregression (SSCA) of CBCL general scales were significant, with a higher effect size for the latter. In order to confirm the validity of each model tested, these models were compared with analogous models in which the path between maternal depression and the CBCL scale was fixed to 0. The differences between the chi-square, computed for each pair of models, corrected with the appropriate formula for MLR parameter estimation [43], were all significant, confirming the relevance of the stable component of maternal depression in the prediction of the CBCL general scales (see Table 5). Interestingly, the unique contribution of the stable component of maternal depression was particularly strong for children’s internalizing symptoms scores, with respect to externalizing and emotional dysregulation ones.

In order to estimate the unique predictive contribution of the stable component of maternal depression on specific CBCL scales, a second set of eight LST models were conducted including the fourth occasion of measurement of each specific CBCL scale (i.e., Anxiety, Withdrawal, Somatization, Social Problems, Thought Problems, Attention problems, Rule Breaking behaviors, Aggressive behaviors) as criterion, and the third occasion of measurement of the corresponding CBCL scale as a covariate. As shown in Table 6, each LST analysis (i.e., 1, 2, 3, 4, 5, 6, 7, 8 models including as criterion respectively Anxiety, Withdrawal, Somatization, Social Problems, Thought Problems, Attention problems, Rule Breaking behaviors, Aggressive behaviors scale) fitted data well, with no significant chi square and adequate levels for the other indices (RMSEA ≤ .05; CFI, TLI ≥ .99; SRMR ≤ .01). The stable component of maternal depression showed significant predictive power on each of the CBCL specific scales at age 12, even when the corresponding CBCL scale at age 8 was controlled for. As shown in Table 6, in each model both the standardized structural coefficient of maternal depression (SSCMD) and the standardized coefficient of autoregression (SSCA) were significant, with higher effect size for the latter. As for previous analyses, to confirm the validity of each solution tested, the models were compared with analogous models in which the path between maternal depressive symptoms and CBCL scale was fixed to 0. The differences between chi-square, computed for each pair of models, corrected with the appropriate formula for MLR parameter estimation [43], were all significant (except for the model that includes Thought Problems as a criterion), confirming the relevance of the stable component of maternal depressive symptoms in the prediction of the CBCL specific scales (see Table 6). The stable-component of maternal depression was particularly predictive of children’s depression scores if compared with the other CBCL symptoms scales. Considering the large number of statistical tests conducted, to test the significance of parameters estimated (i.e., SSCMS, SSCA and Δχ^2^), a correction was applied to conventional alpha level (.05) using Benjamini–Hochberg procedure, but no differences emerged when compared with the conventional testing procedure.

## Discussion

In the global population, identified clinical depressive disorder has a prevalence of 3.4% (affecting more that 260 millions of people), but the number of cases not presenting to clinics, together with individuals suffering non-clinical symptoms, is at least double this figure [1].

In this study, we used a latent state-trait analysis approach (LST) across 6 years (from children’s age 2–8 years) to investigate the possible role of maternal depression stable component in the prediction of general and specific children’s emotional and behavioral symptoms. Interestingly, despite the fact that maternal depression mean scores, as computed across different assessment waves (2, 5, and 8 years of children’s life), tend to decrease overtime, LST analysis showed that individual differences among mothers tend to remain rather stable across 6 years of observation. Further analyses showed that, maternal depression stable component showed a significant contribution in the prediction of each CBCL general and specific scale, as measured at age 12 of the children (except for thought problems scale), even when the corresponding CBCL scale (as measured at 8 years of the children) was controlled for. Interestingly, maternal depression stable component showed a particularly considerable contribution in the prediction of youths’ internalizing symptoms and, in particular of depression. This intergenerational association has previously been shown at the level of maternal disorder [44], but not, to our knowledge, within a community sample using a latent state-trait approach to identify a stable maternal depression component. It is notable that, in the current study, longitudinal assessments were made over 9 years, using four evenly distributed assessment points, in contrast to many previous studies that have used use a narrower framework of observation, generally focusing on early infancy [45, 46]. Other studies, conversely, addressed the effect of maternal depression over long periods of time but with a focus on children’s early childhood and their adolescence, with little if any attention to the intermediate developmental stages (toddlerhood, childhood, latency, early adolescence). The present study, instead, made evaluations over a broad time frame, including the developmental phase around 8 years of age that has been demonstrated to be a critical point in determining future positive or negative outcomes in children’s psychological well being, probably due to an increased capacity of emotion regulation and increased social skills [47–49].

This study had certain limitations. First, we had no data on maternal depression before assessment point 1 (2 years of age of the child). Therefore, we could not know if maternal depression had their onset before or after the child’s birth. This specific piece of information could have been useful to better understand the characteristics of maternal psychopathological risk, and, above all, to understand whether depression began in the peri-partum period. Second, we were unable to obtain data from fathers, and consequently we have no information on the possible role of fathers as a protective factor, or, indeed, as an adjunct risk factor (in case—for instance—of paternal psychopathology) for modulating offspring emotional/behavioral outcome. Third, children’s symptoms were measured through a report form questionnaire, which could suffer from evaluation distortions caused by mothers’ psychopathological state, although this possibility has previously been demonstrated to be minimal (e.g. [27]).

In sum, the current longitudinal analysis of maternal and child data revealed that a stable component of maternal depression significantly predict a wide range of child emotional and behavioral symptoms at 12 years of age, even when the level of those symptoms at age 8 years was controlled for.

## Data Availability

The dataset is visible here: http://dx.doi.org/10.17632/bx62rd3tc2.2.

## Abbreviations

LST: Latent State-Trait
SCL-90/R: Symptom Checklist-90/R
CBCL: Child Behavior Check-List
ANX: Anxiety and depression
WITH: Withdrawal and depression
SOM: Somatization
INT: Internalizing disorders
SOC_PROB: Social problems
TH_PROB: Thought problems
ATT: Attention problems
RU_BR: Rule breaking behaviors
AGGR: Aggressive behaviors
EXT: Externalizing behaviors
EM_DYS: Emotional dysregulation
MDEP: Maternal depression
NM: No method model
NMNO: No occasion and no method factors
MLR: Maximum likelihood estimation robust
SSCMD: Standardized structural coefficient of maternal depressive symptoms
SSCA: Standardized coefficient of autoregression
